# Interference with HIV infection of the first cell is essential for viral clearance at sub-optimal levels of drug inhibition

**DOI:** 10.1371/journal.pcbi.1007482

**Published:** 2020-02-04

**Authors:** Ana Moyano, Gila Lustig, Hylton E. Rodel, Tibor Antal, Alex Sigal

**Affiliations:** 1 Africa Health Research Institute, KwaZulu-Natal, South Africa; 2 School of Laboratory Medicine and Medical Sciences, University of KwaZulu-Natal, Durban, South Africa; 3 School of Mathematics, University of Edinburgh, Edinburgh, United Kingdom; 4 Max Planck Institute for Infection Biology, Berlin, Germany; Temple University, UNITED STATES

## Abstract

HIV infection can be cleared with antiretroviral drugs if they are administered before exposure, where exposure occurs at low viral doses which infect one or few cells. However, infection clearance does not happen once infection is established, and this may be because of the very early formation of a reservoir of latently infected cells. Here we investigated whether initial low dose infection could be cleared with sub-optimal drug inhibition which allows ongoing viral replication, and hence does not require latency for viral persistence. We derived a model for infection clearance with inputs being drug effects on ongoing viral replication and initial number of infected cells. We experimentally tested the model by inhibiting low dose infection with the drug tenofovir, which interferes with initial infection, and atazanavir, which reduces the cellular virion burst size and hence inhibits replication only after initial infection. Drugs were used at concentrations which allowed infection to expand. Under these conditions, tenofovir dramatically increased clearance while atazanavir did not. Addition of latency to the model resulted in a minor decrease in clearance probability if the drug inhibited initial infection. If not, latency strongly decreased clearance even at low latent cell frequencies. Therefore, the ability of drugs to clear initial but not established infection can be recapitulated without latency and depends only on the ability to target initial infection. The presence of latency can dramatically decrease infection clearance, but only if the drug is unable to interfere with infection of the first cells.

## Author summary

A feature of viral infections such as HIV is that successful transmission occurs with low probability and is preventable by administration of drugs before exposure to the virus. Yet, once established, the infection is difficult or impossible to eradicate within its host. In the case of HIV, this may be explained by the establishment of a latent reservoir of infected cells insensitive to antiretroviral drugs. Here we use a combined modelling and experimental approach to determine whether low dose HIV infection can be cleared at drug concentrations which allow the expansion of HIV infection once established. We show that such sub-optimal drug levels are effective at clearing infection, provided they target the virus before it infects the first set of cells. The difference in the effect of drugs before and after the initial cells are infected does not require the establishment of viral latency. Rather, it is a quantitative effect, where the low infection dose can be cleared before amplifying viral numbers by infecting the first cells.

## Introduction

HIV can be suppressed with antiretroviral therapy (ART) to clinically undetectable levels in the blood. However, established HIV infection cannot be cleared with ART, and generally rebounds several week after ART interruption. This persistence is driven by a reservoir of infected cells which decays minimally in the face of ART [1, 2]. There is extensive evidence that a key component of the HIV reservoir is a population of latently infected cells: cells where functional proviral HIV DNA is integrated into the cellular genome but is not expressed [3–6]. Such cells may start producing virus when they are activated [7, 8] and due to stochastic fluctuations in HIV Tat protein production, initiating a positive feedback loop in HIV gene expression [9, 10].

The exception to the failure of ART to clear infection occurs when ART is present during or immediately after an infection attempt. An approach termed pre-exposure prophylaxis (PrEP) aims to administer ART to uninfected, at risk individuals to take advantage of this fact. The majority of clinical studies have shown that PrEP is effective in a variety of populations, transmission modes, and drug delivery modalities [11–19].

The shift from an infection which can be cleared with ART to one which cannot is generally attributed to the formation of the latent reservoir. The early formation of a reservoir of infected cells in the face of ART has been demonstrated in a non-human primate model [20] and latency has been proposed to be a key driver in the initial establishment of HIV infection [21]. While this mechanism is consistent with the very early transition to irreversible infection, it relies on the assumption that ART regimens completely inhibit viral replication in the mucosal tissues of the genital and rectal tracts, the initial HIV infection sites, and that the infection becomes irreversible if the latent reservoir is established before this complete inhibition takes place.

It may be important to consider whether a mechanism which does not rely on the assumption of complete suppression of viral replication in the mucosa with ART, and therefore the rapid formation of a latent reservoir, can lead to this observed behavior of HIV infection. There are several reasons to consider such an alternate: 1) While there is strong evidence that ART levels as measured in the blood are more than sufficient to completely suppress HIV replication [22], drug penetration may be lower in the mucosa. Therefore, whether inhibition is complete in this compartment is less clear [23]; 2) a challenge in PrEP is to maintain adherence to the treatment, as it is administered to uninfected individuals [11–14, 24–26]. If adherence to PrEP is variable, sub-optimal ART concentrations should occur in at least a subset of treated individuals. PrEP was shown to be effective in a non-human primate model of low dose infection even when dosing was intermittent [27], suggesting it may still be effective under conditions of sub-optimal drug; 3) incomplete suppression of viral replication may be relevant to future PrEP approaches [28] which may use agents that have advantages such as long half-lives but do not completely inhibit HIV replication; 4) it may be relevant to understanding basic principles of initial viral infection by using the well characterized HIV infection system which has as a toolkit antiretroviral drugs with different mechanisms of action.

An alternative mechanism would need to explain why, if infection can expand, ART can nevertheless inhibit infection if administered very early after exposure. The alternative hypothesis we propose is that if the initial number of infected cells is small (∼1), it is possible to clear initial infection at sub-optimal inhibitor levels, where such sub-optimal levels would allow infection to expand if the number of initial infected cells was larger. The key conditions are a low initial number of infected cells and an inhibitor which acts before the first cell is infected. The basic reasoning is that under these conditions, the first infected cell is either present or absent. If the inhibitor succeeds in eliminating that infected cell, the infection is cleared regardless of the fact that the infected cell could initiate an expanding infection.

The evidence that a low number of initial infected cells is in fact the physiological condition *in vivo* is that the probability for an individual exposed to HIV by sexual contact to become infected does not exceed 0.02 per sexual act under any set of conditions and is usually much lower [29, 30]. Moreover, infection is established most often with a single viral founder clone [31, 32], and experimental infection with SIV in non-human primates shows the existence of an infection bottleneck at initial infection [33, 34]. These observations indicate that initial transmission is at a low viral dose, sufficient to infect at most one or few cells. This may also be consistent with initial HIV transmission occurring by cell-free HIV infection, where cell-free virions rely on diffusion to reach an infectable cell and therefore have a low probability to infect [35–51]. In contrast, an infected cell is likely to deliver considerable numbers of virions (10^3^ to 10^4^ virions are produced per cell [52, 53]) if it is at close range.

**Fig 1:**
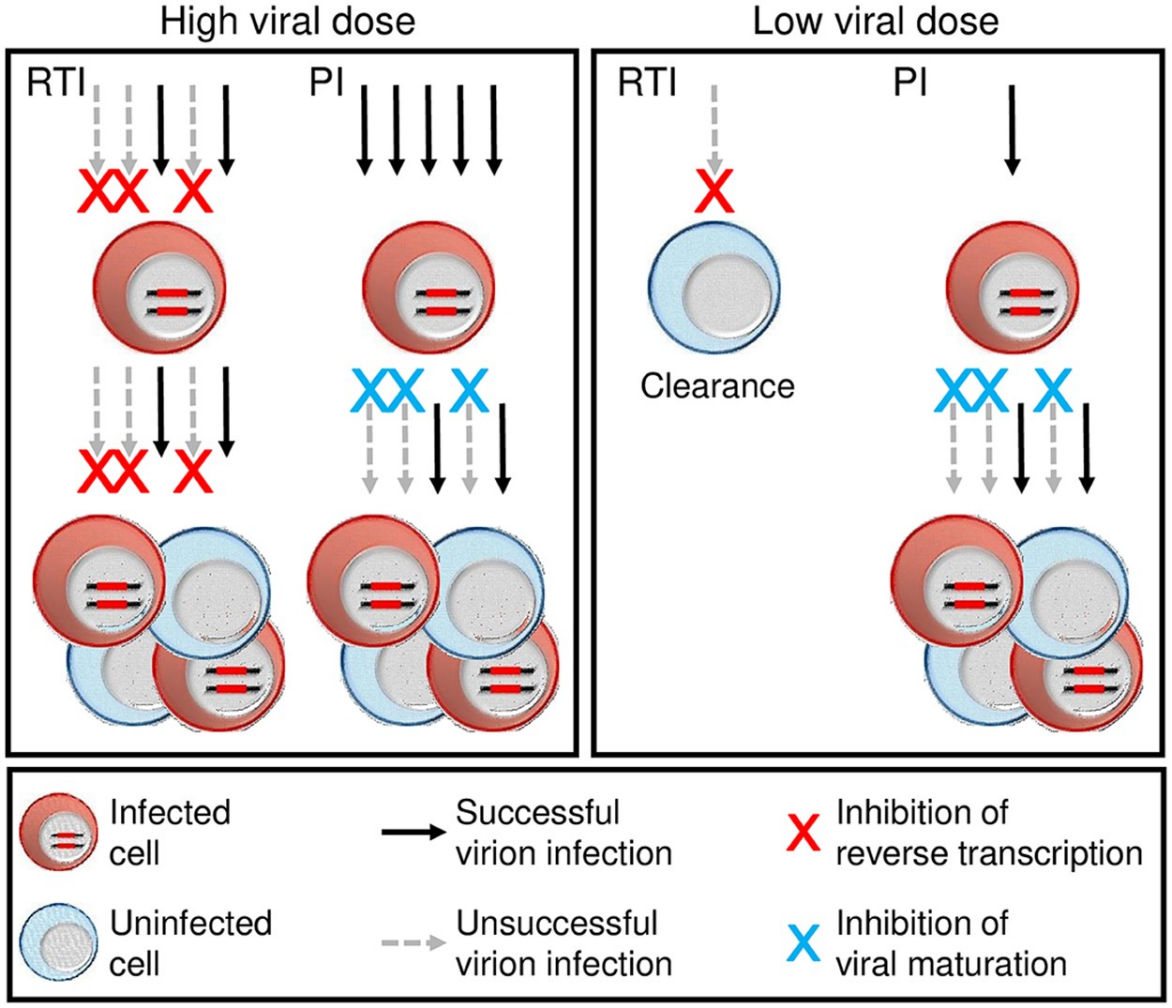
Low dose HIV transmission is vulnerable to clearance before infection of the first cells. Illustrated is partial inhibition of infection (*R*_0_ ∼ 2 with drug) with drugs such as a reverse transcriptase inhibitor (RTI), which acts before HIV integrates into the cellular genome, and a protease inhibitor (PI), which acts after the infection of the first cells by interfering with HIV maturation. Left panel shows high dose transmission between individuals, inhibited by drugs at levels where infection is still able to replicate. Here, the effects of the RTI and PI are symmetrical and neither clears infection. Right panel shows low dose transmission between individuals inhibited at the same drug levels. While the RTI may not clear every infection attempt, it may be successful at clearing infection if the number of infection attempts are few. In contrast, once the first cells are infected, as would occur with the PI, this advantage is lost. In the event *R*_0_ ⪅ 1 with drug, both drug mechanisms can clear infection.

To test whether it is necessary to inhibit before the first infected cells for sub-optimal inhibition to be effective, it is possible to use antiretroviral drugs with different mechanisms of action. HIV reverse transcriptase inhibitors (RTI) such as tenofovir (TFV) prevent the initial infection of the cell but do not interfere with viral production from an already infected cell. That is, they decrease infection frequency. Protease inhibitors (PI) such as atazanavir (ATV) do not interfere with cellular infection but reduce the number of viable mature virions an infected cell produces—the burst size per cell of viable virions. The effect of decreasing infection frequency or viral burst size should be symmetrical at a high viral dose: The number of successful infections will be decreased if fewer virions successfully infect cells or if the ability of infected cells to produce viable virions is reduced (Fig 1, left panel). However, these effects may not be symmetrical at an initial low viral dose (Fig 1, right panel). Since PIs act with a delay—protease mediated cleavage occurs in the virion during budding from an already infected cell—they can be used to study the effects of the delay on the probability of infection clearance with drug when the initial viral dose is only sufficient to infect one or few cells.

Here we tested the hypothesis that the probability of HIV infection clearance with drug levels which allow for viral replication in established infection depends on preventing initial infection of the first one or few cells. We modeled HIV infection as a function of the measurable initial number of infected cells (*N*_0_) and the basic reproductive ratio (*R*_0_)—the number of cells infected on average by one infected cell when infectable cells are not limiting. We then performed experiments with low *N*_0_ and two types of inhibition: reduction of infection frequency by TFV and reduction of viral burst size per cell by ATV. With both drugs, we used a drug concentrations where *R*_0_ > 1 in the presence of drug. That is, infection could expand. We observed that while both drugs reduced *R*_0_ to a similar extent at the concentrations used, only TFV, which prevented successful infection of the first set of cells, was effective at clearing infection.

## Materials and methods

### Ethics statement

The study protocol for blood collection from healthy donors was approved by the University of KwaZulu-Natal Institutional Review Board (approval BE083/18). Blood was obtained with informed written consent from each donor.

### Inhibitors, viruses and cells

The following reagents were obtained through the AIDS Research and Reference Reagent Program, National Institute of Allergy and Infectious Diseases, National Institutes of Health: the antiretroviral drugs ATV and TFV; RevCEM cells from Y. Wu and J. Marsh; HIV-1 NL4-3 CCR5 tropic infectious molecular clone (pNL(AD8)) from E. Freed; pBABE.CCR5, from N. Landau. Cell-free virus was produced by transfection of HEK293 cells with pNL(AD8) using TransIT-LT1 (Mirus) transfection reagent. Supernatant containing released virus was harvested two days post-transfection and filtered through a 0.45 micron filter (GVS). The number of HIV RNA genomes in viral stocks was determined using the RealTime HIV-1 viral load test (Abbott Diagnostics). The RevCEM HIV infection GFP indicator cell line was modified as follows for experiments with the CCR5 tropic virus: The E7 clone was generated from RevCEM cells as described in [36]. Briefly, the RevCEM cell line was sub-cloned by limiting dilution. Clones derived from single cells were expanded into duplicate 96-well plates, one optical and one standard tissue culture for continued growth. The optical plate was infected with HIV strain NL4-3 virus and optical wells were scanned by microscopy to select clones with highest infection percentage by GFP expression. The clone E7 was selected based on greater than 70 percent GFP positive cells upon infection, expanded from the uninfected replicate plate and frozen. To generate the CCR5 expressing B8 reporter clone, RevCEM-E7 cells were infected with the pBABE.CCR5 retroviral vector which stably expressed CCR5 under the LTR promoter. Cells were sub-cloned by limiting dilution. Clones derived from single cells were expanded into duplicate 96-well plates, one optical and one standard tissue culture for continued growth. The optical plate was infected with HIV strain NL(AD8) CCR5 tropic HIV and wells were scanned by microscopy to select clones which maintained similar GFP expression to the parental RevCEM-E7 clonal cell line. The clone RevCEM-B8 was selected based on greater than 70 percent GFP positive cells upon infection, expanded from the uninfected replicate plate, and frozen. Cell culture medium was complete RPMI 1640 supplemented with L-Glutamine, sodium pyruvate, HEPES, non-essential amino acids (Lonza), and 10 percent heat-inactivated FBS (Hyclone).

### Infection and flow cytometry

For determination of drug effect on *R*_0_ and *N*_0_, cells were infected with 2.5 × 10^7^ viral RNA copies in 2ml of cell culture containing 5 × 10^5^ cells/ml. The number of infected cells was acquired every 2 days with a FACSCalibur machine (BD Biosciences) using the 488nm laser line. Flow rate on the machine was measured at each time-point, and acquisition time was multiplied by the inverse of the flow rate to obtain the number of infected cells per milliliter. For experiments measuring $P_{c}^{\text{drug}}$, 200 *μ*l of cells at a density of 5 × 10^5^ cells/ml were infected with 6.3 × 10^3^ viral RNA copies. Results were analyzed using FlowJo 10.0.8 software. The background frequency of positive cells was determined by acquiring uninfected samples (n = 17 from 4 independent experiments). A sample was scored as infected if the number of GFP positive cells was greater than that in the highest background samples (0.01% positive cells).

### Passaging of infected cell cultures

For determination of drug effect on *R*_0_ and *N*_0_, the uninfected and drug treated cell cultures were passaged at a split ratio of 1:2 every 2 days, where half the cell culture was removed and fresh media with drug (for TFV and ATV) or without drug (for uninfected cells) was added. Proliferation of uninfected cells was sufficient to maintain uninfected cell numbers, and infection was below 5 percent for both drug conditions at all time-points, ensuring target cells were not limiting. For the no drug condition, the infection expanded much more rapidly. Therefore, the infected cell culture was passaged by diluting the infected cells 1:100 every 2 days into uninfected cells. Hence, 20*μ*l infected cells were added to 2ml of fresh, uninfected cells at 5 × 10^5^ cells/ml. The removed fraction of cells was used to detect infection by flow cytometry. For experiments measuring $P_{c}^{\text{drug}}$, cell cultures with either no infection or containing ATV or TFV, passaging conditions were the same as for the experiments used to determine drug effect on *R*_0_ except that no culture was removed. Instead, new media with drug was added for the TFV and ATV conditions, and new media with no drug was added for the uninfected condition. The infection volume therefore doubled every 2 days, and the cell culture was transferred to larger volume wells to preserve a constant surface to volume ratio. After 8 days (4 passages), cells were spun down, washed once in medium with no drug, and resuspended at 5 × 10^5^ cells/ml in fresh medium with no drug. Cells were then further passaged in the absence of drug for 6 days (3 passages) using a 1:2 dilution every 2 days to amplify any infection in the culture. For infection in the absence of drug, cells were passaged for 6 days (3 passages) using a 1:2 dilution every 2 days with fresh medium without removing any of the cell culture. The number of infected cells was acquired at the end of the experiment (14 days post-infection for the uninfected, TFV, and ATV conditions, and 6 days for the no drug infection condition) with a FACSCalibur machine as above.

### Measurement of infected cell half-life

For determination of the half-life of infected cells in the presence of ATV, RevCEM-B8 cells were pre-incubated with 16 *n*M ATV for 48h. 10^6^ cells/ml were then infected with NL(AD8) in the presence of ATV to obtain saturating infection (approximately 70 percent GFP positive resulting from 10^9^ viral RNA copies) so that the population of uninfected cells was small and reduction in infected cell number due to cell death could be tracked without the confounding effect of new infections. The cells were maintained with ATV and the number of live infected cells was tracked 2, 4 and 6 days post-infection by pulsing cells with 4 *μ*g/ml of the death detection dye propidium iodide (Sigma-Aldrich) and acquiring for 1 minute with a FACSCalibur machine.

### Measurement of infection clearance in peripheral blood mononuclear cells

Peripheral blood mononuclear cells (PBMCs) were isolated by density gradient centrifugation using Histopaque 1077 (Sigma-Aldrich) and cultured at 2 × 10^6^ cells/ml in complete RPMI 1640 medium supplemented with L-glutamine, sodium pyruvate, HEPES, and non-essential amino acids (Lonza), 10 percent heat-inactivated FBS (GE Healthcare), and IL-2 at 5 *n*g/ml (PeproTech). Phytohemagglutinin at 12 *μ*g/ml (Sigma-Aldrich) was added for 1 day to activate cells. For cell-free infection, PBMCs were pretreated with either TFV, ATV or no drug for 48 hours after activation and before infection. Cells were then infected with 2 × 10^8^ viral RNA copies of NL(AD8) in 1ml of culture. 2 days post-infection, the number of infected cells was determined by fixing and permeabilizing PBMCs using the BD Cytofix/Cytoperm Fixation/Permeabilization kit (BD Biosciences) according to the manufacturer’s instructions. Cells were then stained with anti-HIV p24 FITC conjugated antibody (KC57-FITC, Beckman Coulter, Brea, CA) to detect the presence of intracellular HIV Gag protein. For coculture infection, PBMCs were activated as above. After activation, cells were split into two fractions: donor cells infected with cell-free virus, and target cells to be infected by the addition of the infected donor cells. Donor cells were infected in the absence of drug with 2 × 10^9^ viral RNA copies of NL(AD8) in 2ml of culture. Target cells were incubated with TFV or ATV. 1 day post-donor cell infection, TFV or ATV was added to donor cells. 2 days post-donor cell infection, infected donor cells were stained with carboxyfluorescein succinimidyl ester at 1.5*μ*M (CFSE, Thermo Fisher Scientific) vital stain to differentiate them from target cells, and added to target cells at 1:300 p24-positive infected donor to uninfected target cell ratio. 2 days post target infection, the number of HIV infected, CFSE-negative target cells was quantified by fixing and permeabilizing as for cell-free infection and staining with anti-HIV p24 PE conjugated antibody (KC57-PE, Beckman Coulter, Brea, CA). For determination of clearance probability, 0.5ml of PBMCs at 10^6^ cells/ml were activated as above and pre-incubated with drug for 48h, then infected with 2.5 × 10^5^ viral RNA copies of cell-free NL(AD8). After 2 days, cells were spun down and resuspended into new growth media with drug. After 4 days, cells were washed in 2ml growth media, then resuspended in 0.5 ml media without drug and added to 1.5ml RevCEM-B8 cells at 0.7 × 10^6^ cells/ml to amplify infection. 4 days after addition of PBMCs to RevCEM-B8 cells, the number of infected, GFP positive RevCEM-B8 cells was acquired with a FACSCalibur machine. A sample was scored as infected if the number of GFP positive cells was greater than that in the highest background samples (0.01% positive cells). To approximate *N*_0_ in PBMCs with 2.5 × 10^5^ viral RNA copies, 0.5 ml of PBMC cultured at 10^6^ cells/ml was infected at four virus stock dilutions in triplicate: 1.3 × 10^7^, 6.3 × 10^6^, 3.2 × 10^6^, 1.6 × 10^6^ RNA copies. The number of infected PBMCs was measured after 2 days by flow cytometry using anti-HIV p24 FITC conjugated antibody staining. Infected cell numbers at the viral stock dilutions above were (mean±std): 1.4 ± 0.08 × 10^3^, 6.6 ± 1.9 × 10^2^, 4.7 ± 0.2 × 10^2^, 2.2 ± 0.9 × 10^2^. Data was fit using linear regression to determine *N*_0_, calculated to be approximately 29 infected cells.

## Results

### A model for infection clearance

We first set out to model the effect of drugs on the probability to clear infection (*P*_*c*_). Let *N*_*i*_ be the number of infected cells in the *i*-th transmission step within the newly infected host. The sequence *N*_*i*_, *i* = 0, 1, 2, … is a Markov chain, or, more specifically, a branching process [54] with the random number *N*_*i*+1_ of infected cells at the (*i* + 1)-st infection step determined from the number *N*_*i*_ of infected cells in the previous infection step by the formula

$$\begin{array}{c} N_{i+1}=\sum_{c=1}^{N_{i}}I_{c}. \end{array}$$ (1)

Here *I*_*c*_ are independent identically distributed random variables denoting the number of new cells infected by each infected cell in step *i*. We note that in the case where host cells are not limiting, as occurs in the initial stages of infection, infection chains originating from individual infected cells are independent of each other. The infection is cleared if the number of infected cells *N*_*i*_ becomes zero at any point.

Infection starts with a number of infected cells *N*_0_ as a result of exposure to HIV from an infected individual, where *N*_0_ ≥ 0. *N*_0_ is expected to depend on several factors, among which is the transmitted viral dose during exposure and the cellular infection frequency per virion. *N*_*i*_, where *i* ≥ 1, would then depend on *N*_0_ and the basic reproductive ratio (*R*_0_), the number of cells infected on average by one infected cell in the initial stages of HIV infection, where host cells are not limiting. *R*_0_ depends on both the viral replication rate and the half-life of the infected cells [55, 56] and is approximately 10 *in vivo* [55]. Eventual infection clearance is certain for *R*_0_ ≤ 1. For *R*_0_ > 1, infection may still be cleared if, at any point in the infection chain, the number of infected cells is zero.

To infect new cells, an infected cell produces a burst of *κ* virions, where *κ* is on the order of 10^3^ to 10^4^ [52, 53], and each virion can infect a cell independently with probability *r*. The number of cells infected by a single infected cell in one transmission step has a binomial distribution with mean *R*_0_ = *rκ* [56]. In the biologically relevant case where *κ* is large and *r* is small, with *R*_0_ = *rκ* finite, this binomial distribution can be replaced by the simpler Poisson distribution with mean *R*_0_ [57]. That is, the probability that a single infected cell infects *m* cells (progenies) in one step is $P_{m}=R_{0}^{m}e^{-R_{0}}/m!$.

We denote by *q* the probability that an infection starting from exactly one infected cell is cleared. In this case, it is required that all *m* identical progenies originating from the original infected cell are cleared. Since each progeny is cleared with the same probability *q*, all progenies are cleared with probability *q*^*m*^, assuming independence of progenies. Therefore:

$$\begin{array}{c} q=\sum_{m\geq 0}q^{m}P_{m}. \end{array}$$ (2)

Note that the right hand side of Eq (2) is called the generating function, in this case, of the number of progenies of a single infected cell [54].

Replacing *P*_*m*_ with the Poisson distribution with mean *R*_0_ as described above and using the Taylor series of the exponential function ∑_*m*≥0_ *x*^*m*^/*m*! = *e*^*x*^, we find:

$$\begin{array}{c} q=\sum_{m\geq 0}\frac{q^{m}R_{0}^{m}e^{-R_{0}}}{m!}=e^{R_{0}\left(q-1\right)}. \end{array}$$ (3)

The (smallest non-negative [57]) solution of the above equation gives the probability of clearing the infection for a single initial infected cell:

$$\begin{array}{c} q=-R_{0}^{-1}W\left(-R_{0}e^{-R_{0}}\right). \end{array}$$ (4)

Here, *W* is the Lambert *W*–function [58], the inverse of the function *x* ↦ *xe*^*x*^. The relationship between *q* and *R*_0_ is graphed in S1 Fig, which shows that *q* = 1 for *R*_0_ ≤ 1 and *q* → 0 at *R*_0_ ≫ 1.

Eq (4) derived the probability of infection clearance for exactly one infected cell. The initial number of infected cells may not be one, but may be described as a random variable [21]. We choose it to be a Poisson random variable with mean *N*_0_ which is a biologically relevant distribution in viral infection. Therefore, the probability that the initial number of infected cells is *n* has probability $\phi_{n}=N_{0}^{n}e^{-N_{0}}/n!$. For a fixed number *n* of initial infected cells the infection is cleared with probability *q*^*n*^, assuming infections originating in individual infected cells are independent. To find the probability of infection clearance *P*_*c*_ for a random number of initial infected cells, we take the average over *n*:

$$\begin{array}{c} P_{c}=\sum_{n\geq 0}q^{n}\phi_{n}=\sum_{n\geq 0}\frac{q^{n}N_{0}^{n}e^{-N_{0}}}{n!}=e^{N_{0}\left(q-1\right)}. \end{array}$$ (5)

This is the probability that an infection starting from a Poisson distributed random number of infected cell is cleared, where *q* is given by Eq (4).

We now consider the effect of the antiretroviral drug mechanism on *N*_0_ and *q*. We note that antiretroviral drugs reduce either infection frequency *r* or burst size *κ*. For drugs which reduce infection frequency, *r* → *d*_1_*r*, and for drugs which reduce viral burst size, *κ* → *d*_2_*κ*, where 0 ≤ *d*_1_, *d*_2_ ≤ 1. The no drug case is recovered for *d*_1_ = *d*_2_ = 1. Given *R*_0_ = *rκ* and therefore *R*_0_ → *R*_0_*d*_1_*d*_2_, the effects of the drug mechanisms are symmetrical on *q*

$$\begin{array}{c} q_{\text{drug}}=-{\left(R_{0}d_{1}d_{2}\right)}^{-1}W\left(-R_{0}d_{1}d_{2}e^{-R_{0}d_{1}d_{2}}\right). \end{array}$$ (6)

Hence, if the drugs decrease *R*_0_ to a similar extent, their effect on *q* will also be similar. However, given an initial transmission with cell-free virus, only the drug mechanism that decreases infection frequency will reduce the mean initial number of infected cells *N*_0_. The mechanism which reduces burst size will only affect the success of the next transmission cycle. Therefore, the probability to clear infection with drugs becomes

$$\begin{array}{c} P_{c}^{\text{drug}}=e^{N_{0}d_{1}\left(q_{\text{drug}}-1\right)}. \end{array}$$ (7)

Here *q*_drug_ is determined by Eq (6). The limits for Eq (7) for *R*_0_ ≤ 1 and *R*_0_ ≫ 1 with drug are 1 and $e^{-N_{0}d_{1}}$. At the upper limit for *R*_0_, infection clearance is simply determined by the probability of obtaining *n* = 0 initial infected cells, where the probability to obtain *n* infected cells is a random number from a Poisson distribution with mean *N*_0_*d*_1_. What constitutes a high value for *R*_0_, at which $P_{c}^{\text{drug}}$ only depends on *N*_0_*d*_1_, is discussed below.

**Fig 2:**
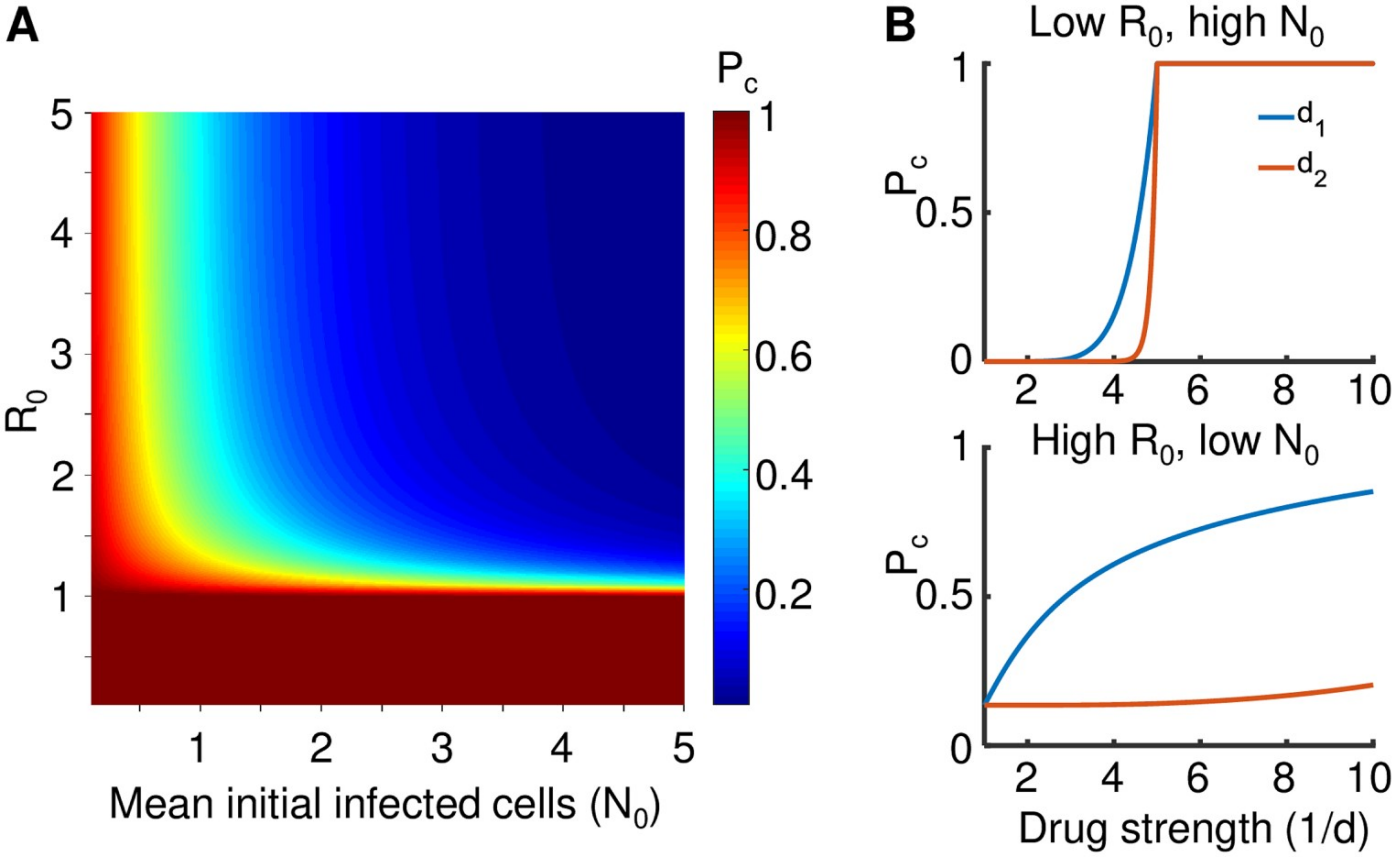
Effects of the initial infected cell number *N*_0_ and *R*_0_ on the probability of infection clearance *P*_*c*_. (A) *P*_*c*_ according to Eq (5) at different parameter values for *N*_0_ and *R*_0_. (B) *P*_*c*_ according to Eq (7) when a drug attenuating infection frequency (*d*_1_, blue line) or burst size (*d*_2_, orange line) acts on *N*_0_ and *R*_0_. Top panel shows the case where *R*_0_ = 5, *N*_0_ = 20, while bottom panel shows the case where *R*_0_ = 20, *N*_0_ = 2. X-axis is drug strength as 1/*d*, y-axis is *P*_*c*_.

To visualize the effects of decreasing *R*_0_ versus *N*_0_, we plotted Eq (5) for a range of parameter values (Fig 2A). It can be observed that for *R*_0_ ≤ 1, infection terminates. At *R*_0_ > 1.5, infection is not strongly sensitive to the exact *R*_0_ value provided *N*_0_ ⪆ 3. However at all *R*_0_ > 1 values, the probability of infection clearance is very sensitive to *N*_0_, provided *N*_0_ is small. This sensitivity is greatly reduced when *N*_0_ ⪆ 3.

To examine the effects of drug mechanism, we plotted infection clearance according to Eq (7) at two conditions of *R*_0_ and *N*_0_ relative to *d*_1_ and *d*_2_ (Fig 2B). In the first condition, *R*_0_ was sufficiently small to be decreased below 1 by the drugs in the inhibition range used, while *N*_0_ was large (Fig 2B, top panel). In the second condition, *R*_0_ was large while *N*_0_ was small (Fig 2B, bottom panel). In the first condition, both drug mechanisms had a similar effect on infection clearance, and *P*_*c*_ = 1 when the effect of either drug reduced *R*_0_ below 1. In the second condition, only *d*_1_, which decreased infection frequency, substantially increased *P*_*c*_. *d*_2_, which acted on burst size, had a minimal effect. We note that based on observations of *R*_0_ ≈ 10 *in vivo* [55] and a probability of infection of at most 0.02 per exposure in the absence of PrEP [29, 30], the second condition likely reflects the physiological situation.

### Experimental determination of the probability of infection clearance with drug

We examined experimentally whether Eq (7) predicts *P*_*c*_ for different drug mechanisms after infection with a low HIV dose, the likely *in vivo* condition for transmission. We used the antiretroviral drugs TFV and ATV to inhibit infection initiating as cell-free HIV. We measured the effect of each drug on the initial number of infected cells *N*_0_ resulting from the initial input of cell-free HIV virions. After this initial cycle of infection, the initial number of infected cells was cultured with uninfected target cells (coculture infection). We define established infection as infection where infected cells are present and can infect new cells using both the cell-free infection route and by cell-to-cell spread [59]. *R*_0_ was measured during this phase of infection.

For virus, we used HIV NL(AD8), an HIV strain with a CCR5 tropic envelope protein. CCR5 tropism has been shown to be the predominant transmitted form between individuals [31]. As target cells for infection, we used a clone of the RevCEM infection indicator cell line [60] which we first subcloned to increase detection efficiency [36] then modified to express the CCR5 receptor (Materials and methods). Detection of infected cells was done by quantifying the number of GFP positive cells using flow cytometry.

We titrated TFV and ATV to obtain a similar effect on ongoing coculture infection. This occurred at 60*μ*M TFV and 16*n*M ATV. To maintain nutrients for cell growth and prevent uninfected cell depletion, we passaged cells every two days (Materials and methods). Such passaging is necessary to maintain conditions where uninfected cells are not limiting in an expanding infection over multiple cell division and viral replication cycles [35].

**Fig 3:**
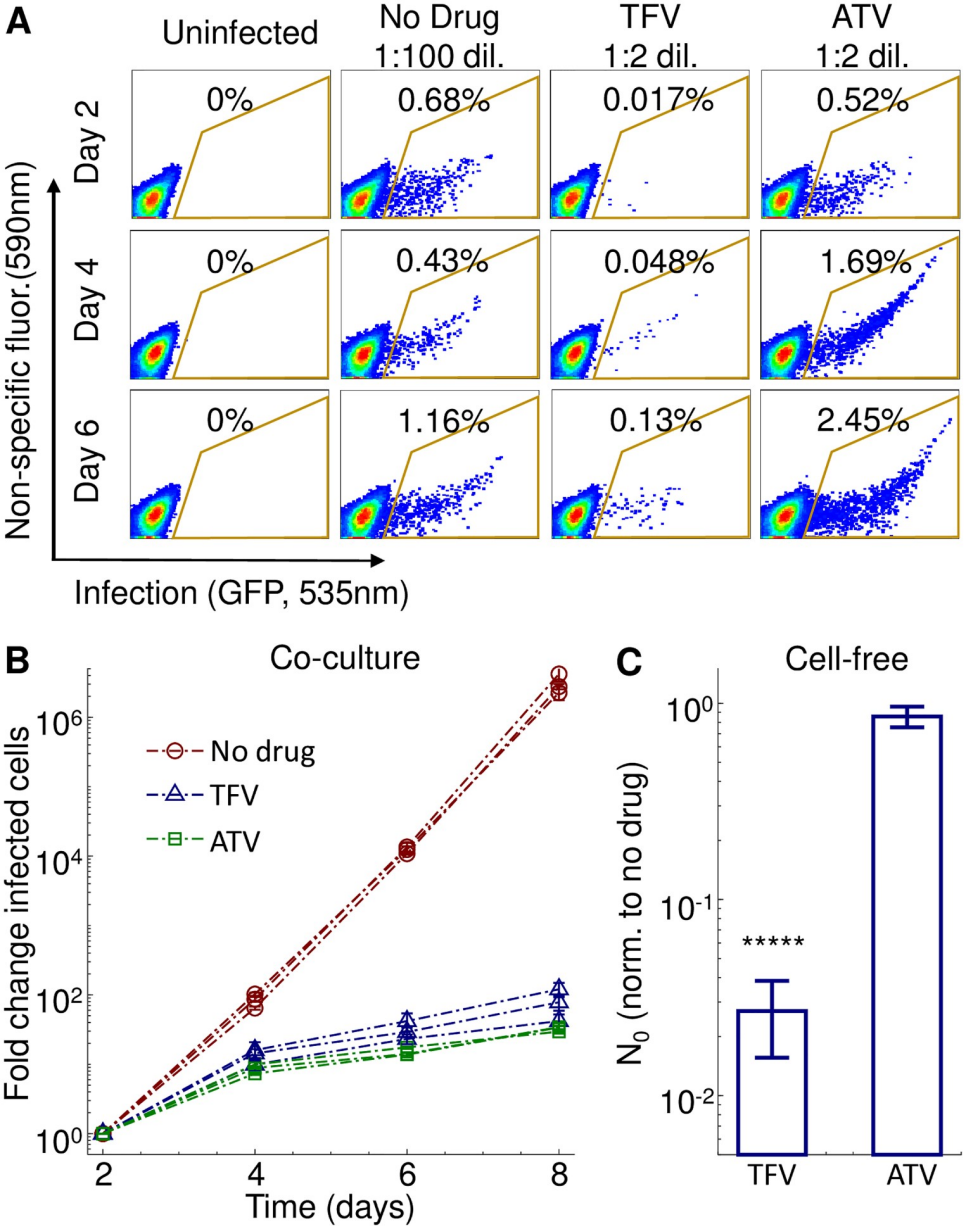
Experimental measurement of drug effect on *R*_0_ and the initial number of infected cells *N*_0_. (A) Flow cytometry plots of the fraction of infected cells at different days post infection in the absence of drug or presence of 60 *μ*M TFV or 16 *n*M ATV. Day 2 is the first time-point after the initial cell-free infection, corresponding to approximately one viral cycle. X-axis is GFP fluorescence, y-axis is autofluorescence, with the fraction of infected cells corresponding to the cells within the area outlined in yellow. Infected cell cultures in the presence of either drug were diluted 1:2 every 2 days. Infected cultures in the absence of drug were diluted 1:100 into uninfected cells every 2 days. (B) Measurement of *R*_0_ in the absence and presence of drug. The number of infected cells at each time-point is normalized by the number of infected cells at day 2 and corrected for the dilution factor used in each infection cycle. 3 independent experiment were performed, with each point denoting the mean ± std of 3 experimental replicates per experiment. Infection in the absence of drug is shown as red circles, TFV as blue triangles, and ATV as green squares. (C) Effect of drug on *N*_0_. For each drug condition *N*_0_ was measured 2 days after cell-free HIV infection and normalized by *N*_0_ for no drug. Mean ± std of 3 independent experiments, where normalization was with *N*_0_ in the absence of drug as measured in the same experiment. Raw numbers of infected cells averaged over all experiments were 1.3 × 10^4^ ± 1.5 × 10^3^ for no drug infection, 3.4 × 10^2^ ± 1.3 × 10^2^ for TFV and 1.1 × 10^4^ ± 7.4 × 10^3^ for ATV (mean ± std). The difference between TFV and ATV was significant (*p* = 6 × 10^−14^ by t-test).

Despite the use of the same HIV cell-free input dose, there were pronounced differences at day 2 between TFV and ATV (Fig 3A). This time-point reflects the results of the initial cell-free infection given an approximately 2 day viral cycle [61]. Cell-free infection was strongly inhibited by TFV relative to no drug. As expected, the effect of ATV on cell-free infection was much weaker since cell-free virus produced in a cell not exposed to a protease inhibitor is already mature. After the day 2 time-point, infection expanded with similar dynamics for both drug conditions, and much more rapidly when no drug was present.

We plotted the total number of infected cells, corrected for cells removed during passaging, versus time (Fig 3B). We then calculated the effect of drug on *R*_0_ over a two day cycle (Table 1). *R*_0_ values showed that infection expanded at a similar rate for the TFV and ATV conditions. We then measured the effect of the drugs on *N*_0_ after the first cycle of infection (day 0 to day 2), and compared the results to infection in the absence of drug. *N*_0_ in the presence of drug divided by *N*_0_ for the no drug condition ($N_{0}^{norm}$) was 0.027 ± 0.014 for TFV and 0.88 ± 0.16 for ATV, (Fig 3C, Table 1). The decrease in *N*_0_ for TFV versus ATV was significant (p = 6 × 10^−14^, t-test).

**Table 1:** Measured parameter values.

Treatment	$N_{0}^{norm}$*	*R*_0_
No drug	1	143 ± 15
60*μM* TFV	0.027 ± 0.014	4.2 ± 0.73
16*nM* ATV	0.88 ± 0.16	3.2 ± 0.088

* *N*_0_ normalized by *N*_0_ no drug.

**Fig 4:**
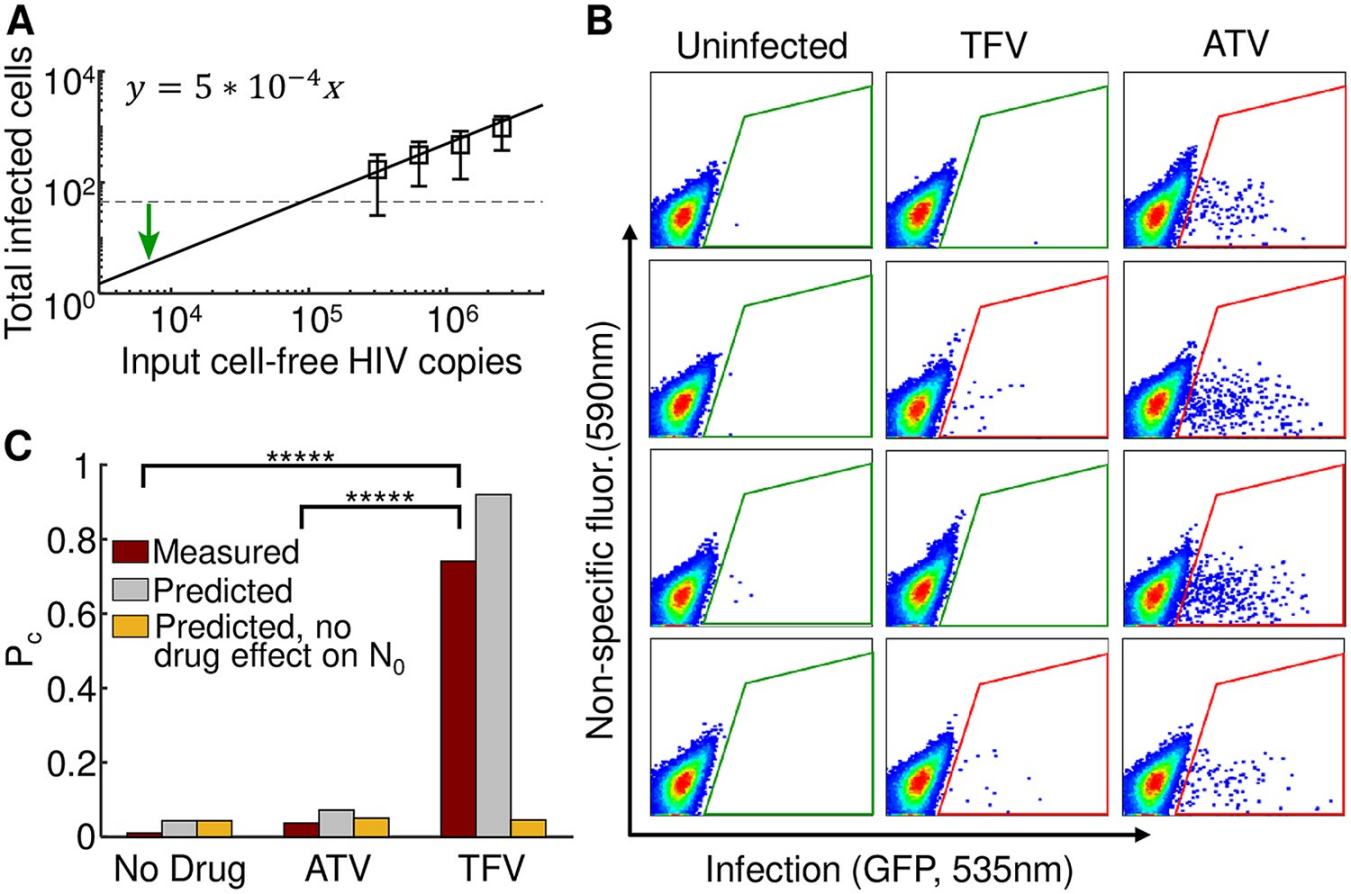
Probability of infection clearance depends on drug mechanism. (A) Determination of *N*_0_. The number of infected cells was measured using flow cytometry as a function of cell-free HIV RNA copies for four virus stock dilutions after one infection cycle (2 days). Data was fit using linear regression to determine the input viral dose for 3 infected cells. Mean ± std of 5 independent experiments. Dashed line is limit of detection. Green arrow marks number of HIV RNA copies used in the experiments. (B) Representative flow cytometry plots after 8 days of infection with the input cell-free virus in the presence of TFV or ATV and further 6 days amplification in the absence of drug. Each plot represents one independently cultured replicate of the experiment. Uninfected samples are shown in the left column, and infection in the presence of TFV or ATV is shown in the middle and right columns respectively. X-axis is GFP fluorescence, y-axis is autofluorescence. The fraction of infected cells corresponds to the cells within the area outlined in green or red, with green indicating background GFP signal level as determined using the uninfected samples, and red indicating above background signal. (C) $P_{c}^{\text{drug}}$ as experimentally measured (red bars), and as predicted by Eq (7) (gray bars) based on the measured drug effects on *R*_0_ and *N*_0_. Presence of infection was assayed in 26 (no drug) or 27 (TFV and ATV) cell-free virus infections from 4 independent experiments. Observed $P_{c}^{TFV}$ was significantly higher than $P_{c}^{ATV}$ and $P_{c}^{Nodrug}$ (*p* = 9 × 10^−8^ and *p* = 5 × 10^−9^ by Fisher’s exact test, respectively). $P_{c}^{ATV}$ and $P_{c}^{Nodrug}$ were not significantly different. Yellow bars show predicted $P_{c}^{\text{drug}}$ if both drugs act on *R*_0_ only. That is, $P_{c}^{\text{drug}}=e^{N_{0}\left(q_{\text{drug}}-1\right)}$.

We then set out to investigate whether TFV and ATV could increase the probability of clearance of low dose infection, corresponding to *in vivo* exposure. We used 6.3 × 10^3^ viral copies (Materials and methods), predicted to result in approximately 3 initial infected cells based on a regression of the number of infected cells versus input viral load (Fig 4A). Infection was initiated with the same cell-free viral dose for all conditions, and infected cells were cultured for 8 days in the presence of drugs. Any infection present was then amplified for detection by culturing cells in the absence of drug. After amplification, infection was either clearly visible or absent (Fig 4B).

We did not experimentally observe clearance of infection in the absence of drug. In the presence of TFV, clearance rose dramatically, with approximately three quarters of infections extinguished. In contrast, only a minor increase of infection clearance was observed with ATV (Fig 4C, red bars). Clearance with TFV was significantly higher relative to no drug and ATV (*p* = 9 × 10^−8^ and *p* = 5 × 10^−9^ by Fisher’s exact test, respectively), while ATV was not significantly different from no drug. Calculation of $P_{c}^{\text{drug}}$ based on Eq (7) using the measured values for *N*_0_ and *R*_0_ for each drug condition replicated an essential feature of the experimental results: treatment with TFV was predicted to result in a much higher clearance probability relative to treatment with ATV (Fig 4C, grey bars). If no effect of drug on *N*_0_ was included in the model, TFV and ATV were predicted to have similar, and small, effects on $P_{c}^{\text{drug}}$ (Fig 4C, yellow bars). Hence, Eq (7) was able to predict the relative effectiveness of each drug to terminate infection.

One explanation for the difference between TFV and ATV clearance frequencies is that the initially infected cells in the presence of ATV were still present at the end of drug treatment due to lack of cell death and gave rise to the infected cell population when ATV was removed. We therefore measured the half-life of cells in the presence of ATV. We observed a half-life of approximately 1 day (S2 Fig). Hence, less than one-tenth of the initially infected cells are expected to survive to the end of drug treatment, making infection persistence with ATV due to a long half-life unlikely.

To examine whether the qualitative pattern of the results obtained for the cell line would also be obtained in primary cells, we repeated the experiment in peripheral blood mononuclear cells (PBMCs) from an HIV uninfected blood donor. PBMCs were infected with a low dose of NL(AD8) strain HIV in the presence of TFV and ATV (Materials and methods). Drug concentrations used were 40 *μ*M for TFV and 24 *n*M for ATV. At these drug concentrations, both drugs reduced infection by approximately one order of magnitude when infection was by coculture of infected with uninfected cells (S3A–S3C Fig), as occurs in established infection. When the infection source was cell-free virus, TFV reduced infection by two orders of magnitude while ATV reduced infection 3-fold (S3D Fig). The reduction with ATV of cell-free infection is consistent with a previous report showing some effect of protease inhibitors on cell-free infection [62], while the greater effect of TFV on cell-free versus coculture infection is consistent with multiple previous studies [35–37, 39, 40, 43, 46, 51]. When infection was with low dose cell-free virus, TFV led to almost complete infection clearance, with no clearance detected for the ATV and no drug conditions (S2E Fig). These results validate the observed behavior of the cell-line infection in primary human cells.

### Effects of latency on the probability of infection clearance

The results above showed that at sub-optimal drug concentrations where HIV infection can replicate, infection can still be cleared if the initial number of infected cells is low and the drug decreases infection frequency before the first cells are infected. This effect does not presuppose the existence of latency. However, given the strong evidence for latency, we investigated the expected effect of latency on infection clearance.

We introduce a probability of a cell to become latent *P*_lat_ [21]. Estimates for *P*_lat_ vary between approximately 0.5 in *in vitro* infections and modelling [10, 63, 64], to 10^−4^ *in vivo*, based on the frequency of intact HIV DNA in the face of ART in CD4+ T cells in the peripheral blood compartment [65, 66], and 10^−3^, based on total HIV DNA copies in rectal CD4+ T cells of individuals on ART [67]. The latter values do not measure the ability of the HIV DNA to produce infectious virus, and therefore the frequency of latent cells containing inducible infectious virus may be lower. However, the value of *P*_lat_ at initial infection is difficult to determine, and therefore values in the upper part of the range cannot be ruled out.

Once a latent cell is produced, the infection may no longer be cleared, since latently infected cells may maintain the reservoir by homeostatic latent cell proliferation as opposed to new rounds of infection [68]. Therefore, infection can persist even if *R*_0_ ≤ 1, provided a latent cell is present.

Infection originating in exactly one initial infected cell clears if it both stays non-latent with probability 1 − *P*_lat_ and independently if all of its progenies clear, which occurs for each of them, again independently, with probability *q*:

$$\begin{array}{c} q=\left(1-P_{\text{lat}}\right)\sum_{m\geq 0}q^{m}P_{m}=\left(1-P_{\text{lat}}\right)e^{R_{0}\left(q-1\right)}. \end{array}$$ (8)

The solution for *q* of Eq (8) is:

$$\begin{array}{c} q_{\text{lat}}=-R_{0}^{-1}W\left(-R_{0}\left(1-P_{\text{lat}}\right)e^{-R_{0}}\right). \end{array}$$ (9)

To account for latency, we simply use *q*_lat_ instead of *q* in Eq (7) to calculate *P*_*c*_.

**Fig 5:**
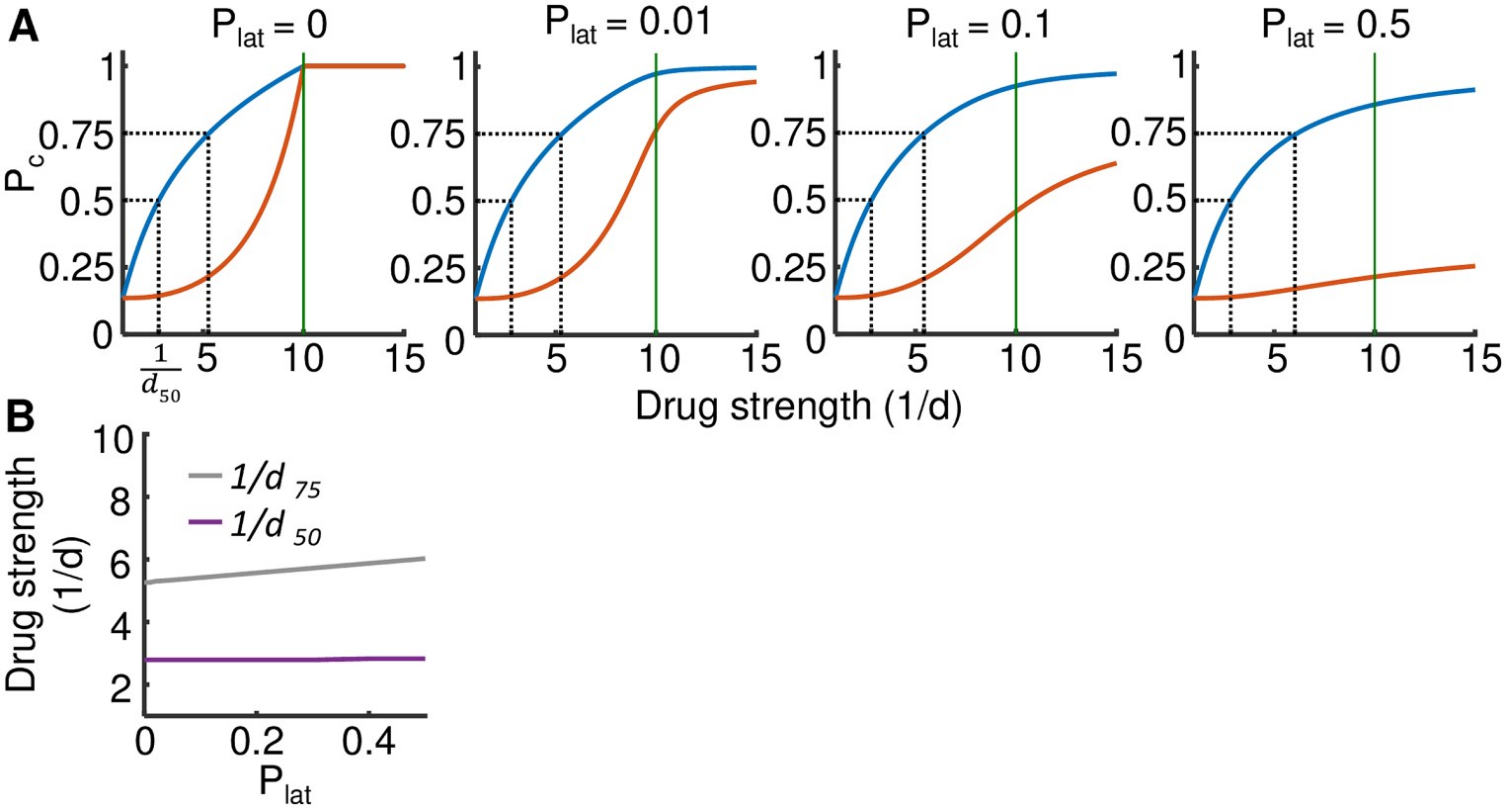
Effect of latency on the probability of infection clearance. (A) *P*_*c*_ was calculated as a function of increasing drug strength (1/*d*) with *N*_0_ = 2 and *R*_0_ = 10. Therefore, at 1/*d* > 10 (denoted by green line), $R_{0}^{\text{drug}}<1$. Drug *d*_1_ (blue line) decreases infection frequency and *d*_2_ (orange line) decreases burst size from an already infected cell. The probability of an infected cell to become latent was *P*_lat_, and the graphs show calculated *P*_*c*_ at the different *P*_lat_ values indicated above each panel. Drug strength for drug *d*_1_ required to clear 50% and 75% of infection attempts are shown by dashed lines. 1/*d*_50_ is indicated in the first panel. (B) Drug strength for drug *d*_1_ required to clear 50% (purple line) and 75% (gray line) of infection attempts as a function of *P*_lat_.

We visualize Eq (9) as the probability to clear infection in the face of increasing drug strength under conditions where the initial number of infected cells is small, while *R*_0_ is within the *in vivo* range for initial infection (*N*_0_ = 2, *R*_0_ = 10, [55]). Therefore, at drug level 1/*d* > 10, $R_{0}^{\text{drug}}<1$ (Fig 5A, horizontal green lines in each graph). We examined clearance with *P*_lat_ ranging from 0 to 0.5 (Fig 5A). We compared the effects on clearance of drug mechanism *d*_2_ which decreases viral burst size, versus *d*_1_ which decreases infection frequency. In the case where *P*_lat_ = 0, *P*_*c*_ = 1 at $R_{0}^{\text{drug}}\leq 1$. As previously described, clearance was lower with mechanism *d*_2_ relative to *d*_1_ at drug levels where $R_{0}^{\text{drug}}>1$ and the difference decreased as $R_{0}^{\text{drug}}\to 1$.

Even at a relatively low frequency of latent cells (*P*_lat_ = 0.01), latency had a visible effect on clearance probability with drug mechanism *d*_2_ (Fig 5A, orange lines). This effect became more pronounced as the frequency of latent cells increased. At *P*_lat_ = 0.5, less than a quarter of infection attempts were cleared at the *d*_2_ drug strength where $R_{0}^{\text{drug}}=1$. In comparison, all infection attempts are cleared at this drug strength without latency. Interestingly, increasing *d*_2_ further increased clearance. This reflects the fact that at higher drug strength, the number of transmission events between cells becomes smaller before the infection terminates. Therefore, the probability of forming a latent cell and hence making infection unclearable becomes lower.

We next examined the sensitivity of drug mechanism *d*_1_ to latency. Unlike with drug *d*_2_, it was difficult to discern the effect of latency on probability of clearance with drug *d*_1_ (Fig 5A, blue lines). We therefore calculated the drug strength necessary to clear 50% or 75% of infection attempts (Fig 5A, dashed lines). Drug strength of drug *d*_1_ required for clearance of 50% of infection attempts was almost unchanged across the range of latent cell frequencies, while drug strength required for 75% clearance increased slightly (Fig 5B). Therefore, in contrast to *d*_2_, drug mechanism *d*_1_ was far less sensitive to the presence of latency, even at the highest frequency of latent cells.

## Discussion

In this study we modeled and experimentally measured the clearance probability of HIV infection as a function of the effect of drug on the basic reproductive ratio of infection *R*_0_ and the number of initial cells *N*_0_ infected by the viral input dose. We chose drug concentrations where HIV infection was able to expand to investigate the effect of sub-optimal HIV inhibition. The reasons to consider sub-optimal drug concentrations are that ART penetration may be lower in the mucosa where the infection takes place, that it is challenging to maintain adherence in healthy individuals on PrEP, and that it is useful to future approaches to understand the basic principles of initial viral infection.

We have shown analytically and experimentally that, under conditions where drugs do not completely inhibit expansion of established infection, it is still possible to clear initial infection provided the number of initial infected cells per infection attempt is low. We derive the clearance probability in Eq (7) and show that clearance is dependent on using a drug which is able to decrease infection frequency and therefore act before the generation of the first infected cells. The intuition is that if *R*_0_ of infection is relatively large despite the drug, termination of infection originating in an initially infected cell becomes unlikely. However, either an initially infected cell is present, or it is not, and the probability of this depends on *N*_0_. If *N*_0_ is low, a drug which can decrease it further will have a strong effect on the probability of infection clearance regardless of its effect downstream of the first infection.

The model output using the measured values for *N*_0_ and *R*_0_ resulted in predicted probabilities of infection clearance which were higher than the experimentally observed clearance frequencies for all conditions. We speculate that this is due to an underestimation of the input number of infected cells *N*_0_. We measured *N*_0_ one viral cycle after cell-free infection. If GFP expression in an infected cell was below threshold of detection at that time, the infected cell would not be detected, yet still amplify infection. Despite this, the relative effectiveness of each drug mechanism was clearly predicted by the model.

Factors *in vivo* which may lead to deviations from model predictions include transmission by cell-to-cell spread [35–51]. Cell-to-cell spread of HIV should reduce the effectiveness of PrEP since the drugs would only act on *R*_0_ and not on *N*_0_. If the initial exposure is indeed to cell-free virus, the higher efficiency of cell-to-cell spread which results in lower drug sensitivity would make sub-optimal levels of ARVs even less likely to be able to clear infection once initial cellular infection has taken place.

In our analysis we assumed that once the first cells are infected, infection proceeds without further bottlenecks and essentially depends on the value of *R*_0_ in the presence of drug. We further considered that a small number of initially infected cells is the physiological situation. Intravaginal SIV infection of rhesus macaques supports the view that the major bottleneck to the establishment of infection is infection of the initial cells. It was observed that even with exposure to a large dose of virus, most of the inoculum was lost at the initial infection stage, and the rest gave rise to few infected cells [33]. Other bottlenecks to systemic infection spread may exist, and establishment of infection may be a two-step process [21], where resting CD4+ T cells are initially infected in the mucosa [33, 69]. HIV is then transmitted with a delay from the mucosa to lymph nodes, a process which may involve transmission of virions on dendritic cells homing to the lymph nodes to present antigen [34, 70]. Therefore, a relatively large number of initially infected cells in the mucosa may decay to one or few infected cells which initiate systemic infection [21]. In this case, it has been shown that the probability to establish infection is ∼*N*_0_*P*_*estab*_, where *P*_*estab*_ is the probability for one initially infected cell to establish infection [21]. Hence, even in a two-step infection process, the sensitivity to *N*_0_ still holds.

HIV has been observed to rapidly seed a latent reservoir of infected cells [20]. We therefore examined the effect of latency on the probability to clear infection as a function of drug strength. Interestingly, for a drug which could target initial infection, clearance probability was similar regardless of whether latency was present or absent. In contrast, latency had a far stronger effect on the probability of infection clearance if the inhibitor used could not interfere with initial infection, even when *R*_0_ < 1. The latter observation is consistent with a critical role for latency in infection establishment under unfavourable conditions for viral replication [21].

The current study shows that sub-optimal drug inhibition can clear HIV infection before it is established, provided the number of initial HIV infected cells is low, and the drug is able to target initial infection. In this situation, the presence or absence of latency has a weak impact on the outcome. More generally, it indicates that in diseases which involve transmission of low pathogen numbers upon exposure, but have robust replication when established, a possibility to clear infection should exist even with relatively weak inhibition if initial infection is targeted.

### Conclusion

We investigated why initial HIV infection can be cleared with inhibitors before it is established but not after. We modelled infection with a branching process and used *in vitro* experimentation to test the model. We examined two drug mechanisms: inhibition of infection frequency, and reduction of the burst size of viable virions from an already infected cell. We found that the small difference in timing between the two mechanisms is critical in clearing of low dose HIV transmission. Despite similar effects of both drug mechanisms on HIV replication, only the drug mechanism reducing infection frequency, which could act before the first cells were infected, was able to clear infection. We conclude that the difference may not require the presence of a latent reservoir, but is rather a numbers game: while an imperfect drug may not clear every infection attempt, it may be successful at clearing infection if the number of cellular infection attempts are few.

## Supporting information

S1 Fig *q* as a function of *R*_0_. (TIF)

S2 Fig Estimation of the half-life of infected cells in the presence of ATV. Half-life of infected cells was estimated using the fraction of live infected cells over time in the presence of ATV after saturating infection. Shown are the means and standard deviations of the number of live infected cells normalized by the number at the first time-point measured. Line is the fit to *y* = *e*^*rt*+*b*^, with *r* = -0.66/day. Half-life was 1.05 days.
(TIF)

S3 FigInfection clearance with drugs in primary cells.(A) Gating strategy to detect the number of infected cells in coculture infection. Cells were first infected with cell-free HIV and used as the infecting (donor) cells for coculture infection. Donor cells were labelled with CFSE and added to uninfected target cells (Materials and methods). To quantify the number of infected target cells, the lymphocyte population was selected using forward scatter (FSC) and side scatter (SSC) and donor cells were gated out by selecting the CFSE negative population. (B) Fraction of infected target cells in coculture infection. X-axis shows infection as detected using a stain for intracellular HIV Gag protein, y-axis is CFSE fluorescence. First plot shows uninfected cells, second plot shows infection in the absence of drug, third plot shows infection with 24 *n*M ATV, and forth plot is infection with 40 *μ*M TFV. (C) Decrease in coculture infected target cells with drug relative to no drug with 40 *μ*M TFV or 24 *n*M ATV. *Tx* = (number infected cells with drug)/(number infected cells without drug). Mean and standard deviation of 3 replicates from two independent experiments. (D) Decrease in the number of cell-free infected cells with drug relative to no drug (Tx, equivalent here to $N_{0}^{norm}$) with 40 *μ*M TFV or 24 *n*M ATV. (E) Probability of infection clearance with 40 *μ*M TFV or 24 *n*M ATV. Pooled data from 5 independent experiments, *n* = 45 samples each for no drug, TFV, and ATV. None of the infection attempts with no drug or ATV were cleared, while all but 2 of the infection attempts were cleared with TFV. Difference between TFV and the other two conditions was significant (*p* = 2 × 10^−23^ by Fisher’s exact test).(TIF)
