# Supplementary figures and images for: Interference with HIV infection of the first cell is essential for viral clearance at sub-optimal levels of drug inhibition
Source: PLoS Comput Biol. 2020 Feb 4;16(2):e1007482. doi: 10.1371/journal.pcbi.1007482 (PMC7039526; doi:10.1371/journal.pcbi.1007482)

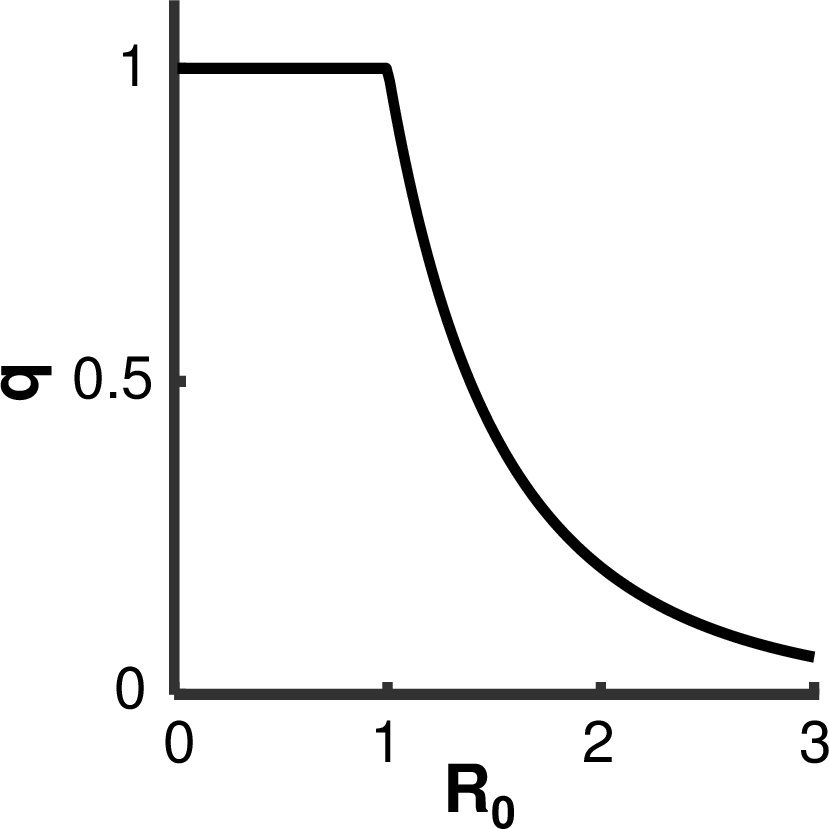

Supplement: S1 Fig — (TIF) [file pcbi.1007482.s001.tif]

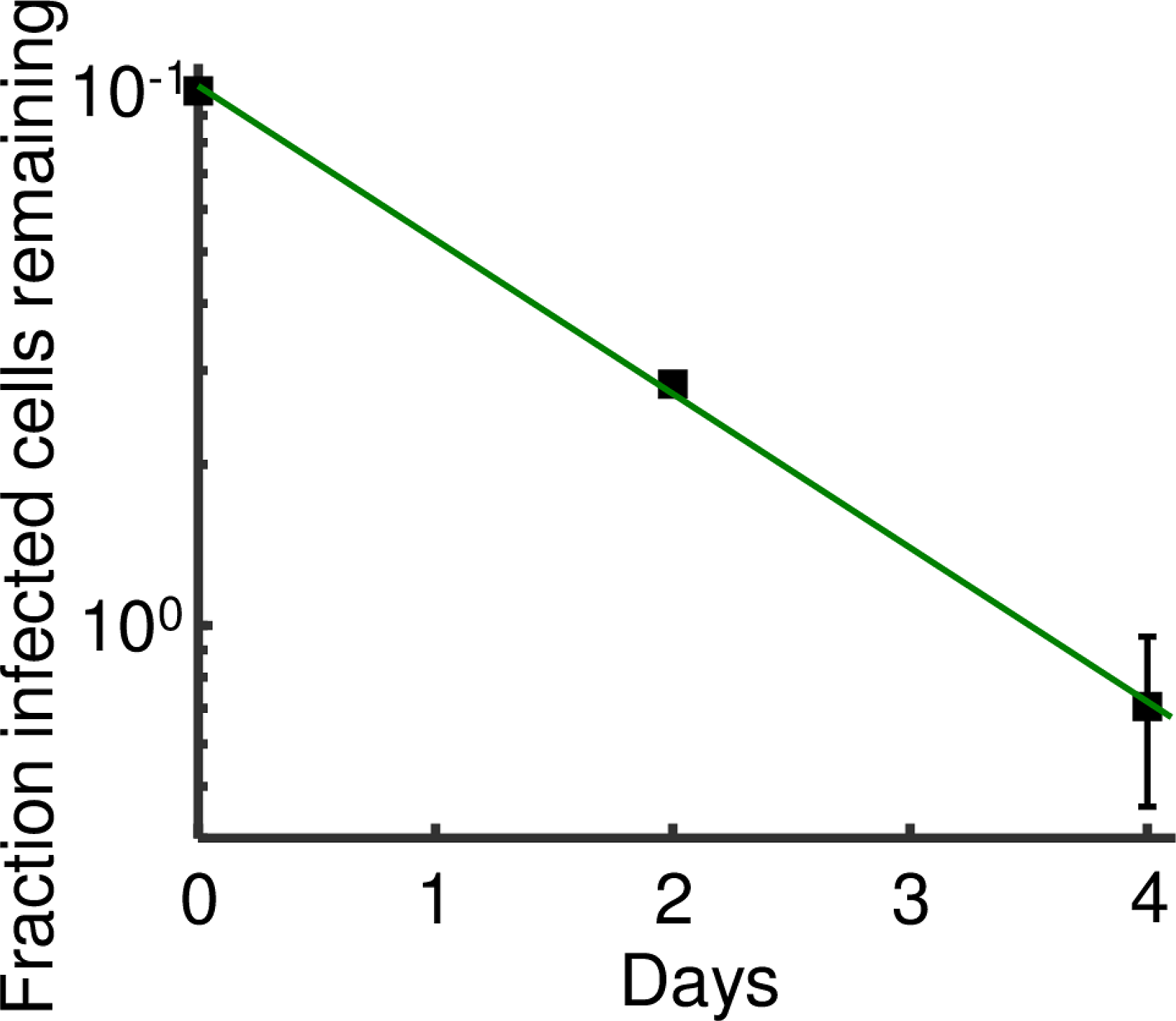

Supplement: S2 Fig — Half-life of infected cells was estimated using the fraction of live infected cells over time in the presence of ATV after saturating infection. Shown are the means and standard deviations of the number of live infected cells normalized by the number at the first time-point measured. Line is the fit to y = ert+b, with r = -0.66/day. Half-life was 1.05 days. (TIF) [file pcbi.1007482.s002.tif]

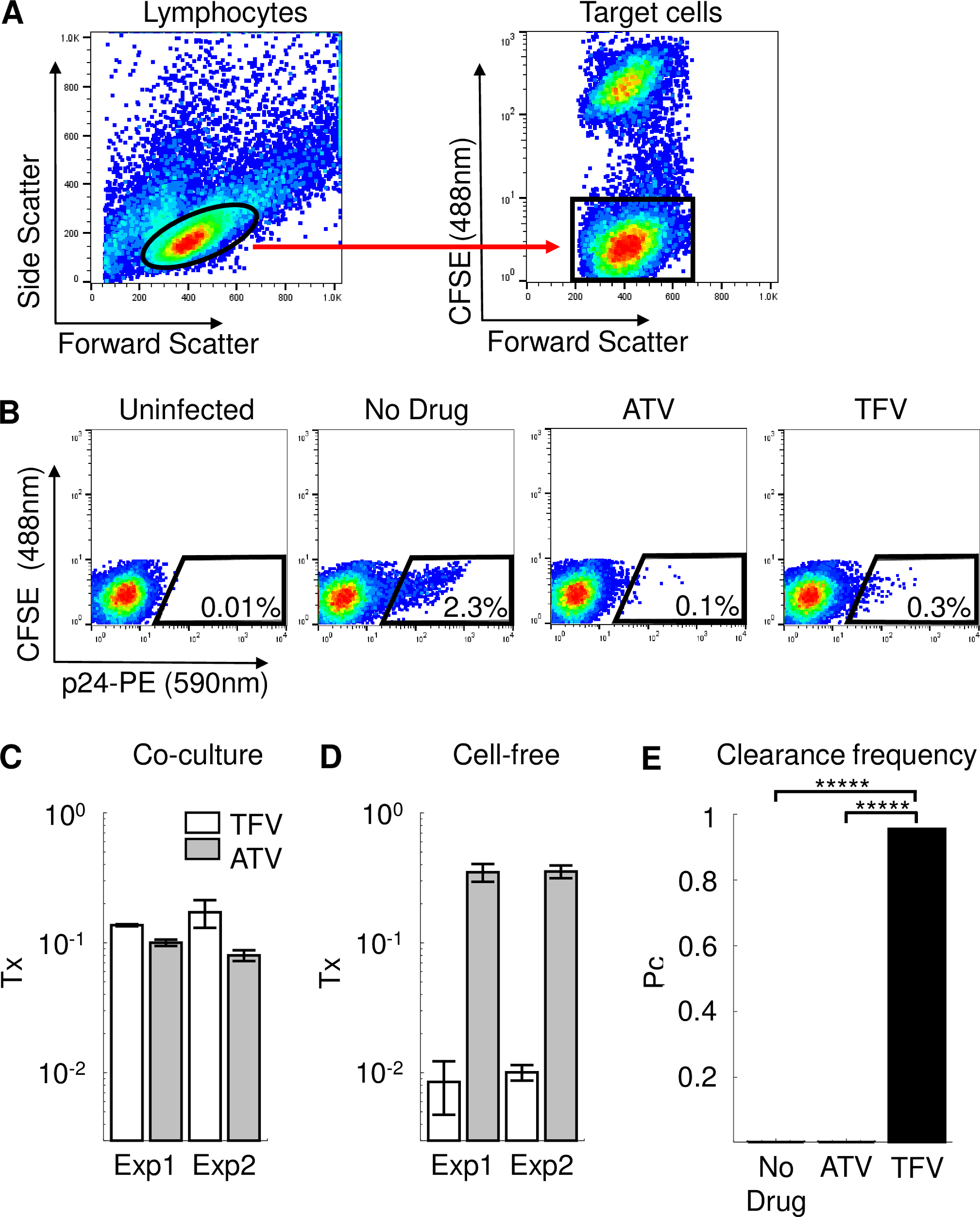

Supplement: S3 Fig — (A) Gating strategy to detect the number of infected cells in coculture infection. Cells were first infected with cell-free HIV and used as the infecting (donor) cells for coculture infection. Donor cells were labelled with CFSE and added to uninfected target cells (Materials and methods). To quantify the number of infected target cells, the lymphocyte population was selected using forward scatter (FSC) and side scatter (SSC) and donor cells were gated out by selecting the CFSE negative population. (B) Fraction of infected target cells in coculture infection. X-axis shows infection as detected using a stain for intracellular HIV Gag protein, y-axis is CFSE fluorescence. First plot shows uninfected cells, second plot shows infection in the absence of drug, third plot shows infection with 24 nM ATV, and forth plot is infection with 40 μM TFV. (C) Decrease in coculture infected target cells with drug relative to no drug with 40 μM TFV or 24 nM ATV. Tx = (number infected cells with drug)/(number infected cells without drug). Mean and standard deviation of 3 replicates from two independent experiments. (D) Decrease in the number of cell-free infected cells with drug relative to no drug (Tx, equivalent here to N0norm) with 40 μM TFV or 24 nM ATV. (E) Probability of infection clearance with 40 μM TFV or 24 nM ATV. Pooled data from 5 independent experiments, n = 45 samples each for no drug, TFV, and ATV. None of the infection attempts with no drug or ATV were cleared, while all but 2 of the infection attempts were cleared with TFV. Difference between TFV and the other two conditions was significant (p = 2 × 10^−23^ by Fisher’s exact test). (TIF) [file pcbi.1007482.s003.tif]
